# An eHealth Intervention to Increase Physical Activity and Healthy Eating in Older Adult Cancer Survivors: Summative Evaluation Results

**DOI:** 10.2196/cancer.6435

**Published:** 2017-03-01

**Authors:** Paul Krebs, Jonathan Shtaynberger, Mary McCabe, Michelle Iocolano, Katie Williams, Elyse Shuk, Jamie S Ostroff

**Affiliations:** ^1^ Department of Population Health New York University School of Medicine New York, NY United States; ^2^ Cancer Survivorship Initiative Memorial Sloan Kettering Cancer Center New York, NY United States; ^3^ Memorial Sloan Kettering Cancer Center Department of Psychiatry and Behavioral Science New York, NY United States

**Keywords:** survivors, diet, food and nutrition, breast neoplasms, prostatic neoplasms, eHealth

## Abstract

**Background:**

A healthy lifestyle is associated with improved quality of life among cancer survivors, yet adherence to health behavior recommendations is low.

**Objective:**

This pilot trial developed and tested the feasibility of a tailored eHealth program to increase fruit and vegetable consumption and physical activity among older, long-term cancer survivors.

**Methods:**

American Cancer Society (ACS) guidelines for cancer survivors were translated into an interactive, tailored health behavior program on the basis of Social Cognitive Theory. Patients (N=86) with a history of breast (n=83) or prostate cancer (n=3) and less than 5 years from active treatment were randomized 1:1 to receive either provider advice, brief counseling, and the eHealth program (intervention) or advice and counseling alone (control). Primary outcomes were self-reported fruit and vegetable intake and physical activity.

**Results:**

About half (52.7%, 86/163) of the eligible patients consented to participate. The most common refusal reasons were lack of perceived time for the study (32/163) and lack of interest in changing health behaviors (29/163). Furthermore, 72% (23/32) of the intervention group reported using the program and most would recommend it to others (56%, 14/25). Qualitative results indicated that the intervention was highly acceptable for survivors. For behavioral outcomes, the intervention group reported increased fruit and vegetable consumption. Self-reported physical activity declined in both groups.

**Conclusions:**

The brief intervention showed promising results for increasing fruit and vegetable intake. Results and participant feedback suggest that providing the intervention in a mobile format with greater frequency of contact and more indepth information would strengthen treatment effects.

## Introduction

More than 13.7 million persons in the United States have a history of cancer, a number that has been steadily increasing due to progress in detection and treatment and the overall aging of the population [1]. Most cancer survivors are aged above 55 years and are at increased risk for comorbid conditions, such as cardiovascular disease and diabetes. Many also experience long-term negative effects of treatment such as fatigue, cognitive impairment, pain, and reduced health-related quality of life (HRQoL) [2-4].

Adopting healthy lifestyle behaviors such as physical activity and eating a diet high in fruits and vegetables can improve HRQoL [5,6]. The American Cancer Society (ACS) and an expert panel of the American College of Sports Medicine (ACSM) suggest that survivors should aim to exercise at least 150 min per week and engage in muscle-strengthening activities at least 2 days per week [7] *.* In addition, the ACS recommends a dietary pattern that is high in vegetables, fruits, and whole grains [8]. Nonadherence to these behaviors also leads to being overweight or obese, which may independently increase the risk of recurrence for colon, prostate, and breast cancers [9-11].

Although survivors indicate interest in dietary and physical activity improvements, several studies have found that a diagnosis of cancer does not necessarily result in increased adherence to healthy lifestyles. Despite the potential benefits of being physically active, 75% of cancer survivors report not engaging in the recommended 150 weekly min of at least moderate physical activity [12] and more than 80% of cancer survivors are not meeting daily fruit and vegetable intake recommendations [6].

Findings from a national population survey of cancer survivors have suggested a need to intervene on more than one behavior to improve HRQoL among survivors [6]. Optimizing health behaviors, however, remains a challenge in the health care setting. The majority (70-80%) of survivors report that health care providers have not discussed physical activity or healthy eating with them [13,14] even though survivors prefer to receive such counseling within the health care setting [15,16] *.* Although several health behavior change interventions have been created and found to be efficacious for fruit and vegetable consumption and exercise [17], most of them have relied on in-person and telephone-based counseling modalities [17-20] creating challenges for widespread adoption by health care settings on a long-term basis [21]. eHealth behavior change interventions can reduce many implementation barriers [22,23] and thereby reach a greater number of survivors. Thus, our team worked with clinicians (nurse practitioners in a designated Survivorship Clinic) to develop and pilot a multibehavior change intervention for adult cancer survivors that would be easily disseminable and sustainable. A digital video disc (DVD) format was chosen, similar to other interventions for older adults, [24,25] as mobile phone and Internet access remains lower among older populations and was particularly so at the time (only 58% of our target population had access to either) [26,27]. The goal of the project was to provide information on feasibility and modifications needed for a larger trial. This paper reports recruitment data and summative evaluation outcomes of the intervention collected at the final assessment.

## Methods

### Participants and Procedures

Patients at Memorial Sloan Kettering Cancer Center (MSKCC) who had completed their primary treatment for either breast or prostate cancer no more than 5 years previously and had an intake scheduled at the Survivorship Clinic were identified via the clinical database and sent an invitational letter signed by the director of the clinic describing the study and procedures. Two weeks later they were contacted by telephone. Once contacted, they were screened for additional eligibility criteria: the presence of at least one behavioral risk factor (engaging in <150 min of physical activity per week or eating less than 5 fruits or vegetables per day), English-speaking, and able to provide informed consent. During this call, the research assistant answered any questions about the study and scheduled a time to meet the patient immediately before their first Survivorship Clinic appointment to complete informed consent, the baseline survey, and randomization. Patients completed the survey on their own. The research assistant was present to clarify any questions when needed. Following the survey completion, the research assistant contacted the research office who provided the group assignment using the permuted block method stratified by the disease type (breast or prostate). If randomized to the intervention arm, the research assistant gave the participant the DVD and explained how to view it. One month following the clinic appointment, patients in the intervention arm were mailed a reminder letter to use it. Three months following the clinic appointment, all patients were mailed follow-up surveys and given the option to complete the surveys by phone or mail. At the completion of the study, patients in the control arm were offered the intervention components. The study was approved by the MSKCC and New York University Medical Center Institutional Review Boards. As a pilot, it was not a registered trial.

### Experimental Conditions

#### Intervention

On the basis of formative evaluation data with patients and providers, the intervention was provided on DVD as this modality, compared with mobile phones or Internet access, was most available to the older adult population at the time [26,28]. The intervention was guided by Social Cognitive Theory [29] and contained components of prior evidence-based interventions developed for cancer survivors [30]. This included focus on enhancing knowledge about the behaviors, developing positive expectancies, reducing barriers, supporting self-efficacy, and stories from cancer survivors. The program provided specific dietary and physical activity recommendations which were drawn from the ACS guidelines for cancer survivors [8]. These focused on eating at least five or more servings of fruits and vegetables a day, choosing high-fiber breads and cereals, lean protein, and low-sugar unprocessed products. For activity, the recommendation was to get at least 30 min of moderate to vigorous activity a day, and at the very least focus on reducing sedentary time. Suggestions were provided for how to change behaviors. For instance, for dietary behaviors the DVD had them choose a healthy eating goal for the next week and provided general tips such as “focus more on benefits than losses,” “keep track of progress,” “set small goals,” and “get family or friends involved” along with a detailed voiceover narration about how to carry out each of these. A focus group of 9 clinicians with expertise in cancer survivorship (nurse practitioners, medical oncologists, clinical nutritionist, and health psychologists) reviewed a draft of the intervention and made suggestions for optimizing structure and content. The intervention made use of branching menus (accessed using a computer or DVD player remote control), which were used to tailor information and feedback on the following variables: level of activity and dietary adherence, readiness to change, barriers, benefits, knowledge, and goal setting (Multimedia Appendix 1). For instance, users could choose which barriers to healthy eating they wanted to hear more about (eg, getting family to eat vegetables, reducing food waste with fresh food, or feeling full), which was then followed by a description from a clinical nutritionist of options for overcoming each barrier. In addition, clips of interviews with 6 survivors were interspersed throughout the program to emphasize particular themes and provide opportunity for identification and modeling. Each topic (healthy eating and physical activity) was divided into 4 chapters each (for a total of 8 sections): (1) importance of the healthy behavior for survivors, (2) self-assessment, (3) behavior change strategies, and (4) links to additional information. The total DVD including healthy eating and physical activity took about 60 min to complete but could range from 45 to 90 min, depending on the participant’s choice of branching menus. Instructions were provided by the research assistant and the DVD jacket also contained technical instructions for how to play it as well as how to make use of it noting they could “choose whatever sections you are interested in and go back and review them as much as you want.”

#### Control

The control group received standard care at the MSKCC Survivorship Clinic, which consists of routine health behavior assessment and advice and brief counseling regarding health maintenance provided by a nurse practitioner with expertise in cancer survivorship.

### Measures

#### Fruit and Vegetable Intake

Fruit and vegetable intake was measured by the Thompson Food Frequency Questionnaire [31], which assesses quantity of food consumption by meal and computes a score on the basis of the total consumption of each food category. The measure defined servings of each food according to standards published by the US Department of Agriculture [32]. For fruit consumption, daily servings can range from 0 to 4.5. For vegetable consumption, servings can range from 0 to 6.75. To compute combined fruit and vegetable consumption scores, the 2 scores were summed together with a total score ranging from 0 to 11.25. The measure has been used in numerous studies and has found to be correlated with intake for older women (.53) and men (.67) [32].

#### Physical Activity

The Godin Leisure-Time Exercise Questionnaire [33] was used to assess physical activity. The questionnaire asks participants to report their weekly performance of minutes spent engaged in mild, moderate, and strenuous exercise. The reported frequency of the various types of exercise is then converted into Metabolic Equivalent of Task units (METs). METs were computed by multiplying each reported instance of mild physical activity by 3, moderate activity by 5, and strenuous activity by 9 [33]. The measure has been found to have similar validity to other self-report measures and found to be correlated with accelerometer data in breast cancer survivors (.53) [34].

#### Demographics

Participants reported their age, sex, race, marital status, highest education, occupation, and income. Primary cancer diagnosis was extracted from the medical record.

### Qualitative Patient Feedback

All intervention group participants completed use and evaluation items at the 3-month follow up [35]. Qualitative interviews (n=12) were also conducted with a random sample of intervention group participants who used the intervention. These were used to further investigate the acceptability and feasibility of the intervention and to inform improvements to future iterations of the program. The interviews were conducted over the phone by a qualitative methods specialist (Ms Shuk) and were limited to 45 min. Audio recordings were transcribed by an independent transcription company (RA Fisher, Inc).

### Analytic Plan

The primary goal of this pilot study was to examine patient interest in and feasibility of the intervention in order to guide the development of a larger trial. We therefore detail screening, exclusion, and refusal reasons. For each primary outcome, we report means and standard deviations at baseline and 3-month follow up along with effect sizes (Cohen d). This was calculated as difference in the change scores for intervention versus control divided by the pooled standard deviation. For dietary intake, we reported the number of fruit servings, vegetable servings, and combined fruit and vegetable servings. Between-group differences were not analyzed as the pilot was not powered to detect statistically significant differences. All analyses were conducted in SAS version 9.3 (SAS Institute, Inc).

The qualitative data were reviewed using inductive thematic text analysis, an iterative process of transcript review, interpretation, and consensus discussions [36-38]. An initial set of 3 interview transcripts were coded by 2 independent reviewers (Ms Shuk and Ms Williams), in which each reviewer read the same transcript, highlighting important content and recording reflections on the transcript in a process known as margin coding [39], prior to completing a written analysis template with supporting participant quotations. The reviewers subsequently met to generate collective findings for the transcript. Once key thematic findings had been identified for the first 3 transcripts, the reviewers subsequently read and coded the remaining transcripts through the same process, both exploring the themes that had been established and identifying additional salient findings. As per standard procedures, the final analytic phase entailed generating higher-order descriptive and interpretive themes that represented the most frequent concepts observed across all interviews.

## Results

### Participants

**Figure 1:**
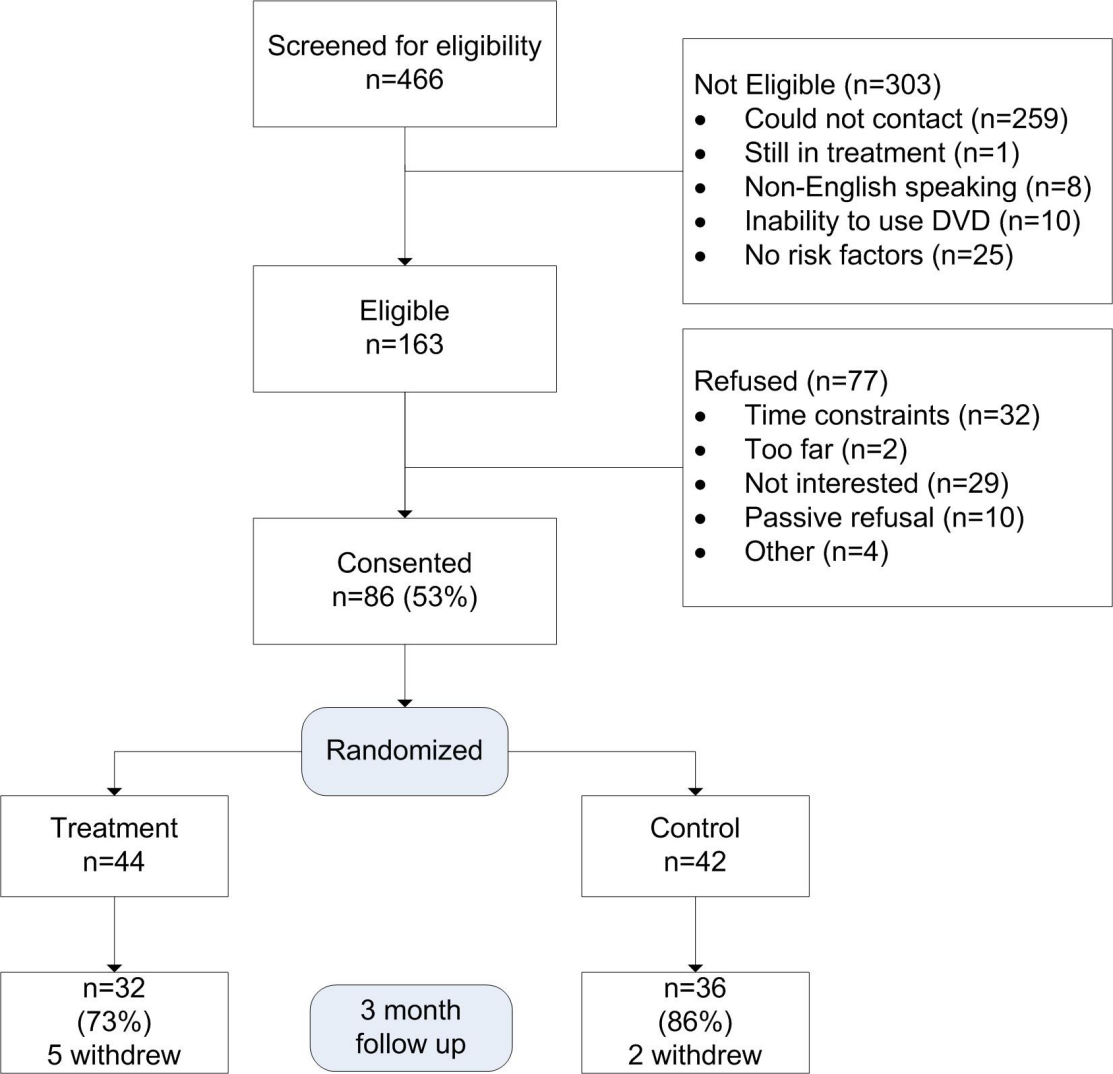
Patient Flow.

A total of 466 individuals were screened for eligibility. Of those, 259 could not be contacted via phone and 25 had no risk factors. Additionally, 10 individuals reported inability to operate a DVD, 8 were non-English speaking, and 1 was still under treatment. Of the 163 eligible individuals, 86 consented to participate for a 52.7% participation rate. The main reasons for refusal were time constraints (n=32) and lack of interest in making health behavior changes (n=29). At the 3-month follow up, the retention rate was 73% (32/44) and 86% (36/42) for the intervention and control groups, respectively (Figure 1). Recruitment and data collection were conducted from August 2013 to March 2014.

Demographic characteristics of the sample are shown in Table 1. Participants were predominantly non-Hispanic white (81%, 69/86), and female (96%, 82/86), with a mean age of 59.8 (standard deviation, SD 11.4). Recruitment of prostate cancer survivors was limited due to clinic scheduling and change in staffing during the study period.

**Table 1:** Demographic Characteristics (n=86).

Demographic characteristics^a^		n (%)
**Age in years,** (mean 59.8, SD 11.4)		
	35-54	29 (34)
	55-64	25 (30)
	65-74	20 (23)
	75+	11 (13)
**Sex**		
	Female	82 (96)
**Primary cancer diagnosis**		
	Breast	83 (97)
	Prostate	3 (3)
**Relationship status**		
	Married or partnered	59 (69)
**Race or ethnicity**		
	Non-Hispanic white	69 (81)
	Non-Hispanic black	5 (6)
	Non-Hispanic Asian	2 (2)
	Non-Hispanic other	2 (2.4)
	Multiracial	2 (2)
	Hispanic	5 (6)
**Employment status**		
	Employed	41 (48)
	Homemaker	7 (8)
	Retired or disabled	35 (38)
	Unemployed	2 (2)
**Education**		
	≤High school	7 (8)
	Some college	21 (25)
	College graduate	18 (21)
	Graduate degree	39 (46)
**Income (K)**		
	10-29	4 (5)
	30-49	7 (8)
	50-69	12 (14)
	70-89	14 (17)
	90k+	47 (56)

^a^One person consented but did not choose to complete demographic data. Two people did not complete income data.

### Primary Outcomes

#### Fruit and Vegetable Consumption

As shown in Table 2, the intervention group increased its intake by 0.18 servings whereas the control group decreased their intake by 0.10 (d=0.25). Fruit and vegetable intakes were also analyzed separately. The mean fruit score at follow up increased by 0.09 for the intervention group and decreased 0.08 for the control group (d=0.33). The vegetable score increased to 0.08 for the intervention group and decreased to 0.02 for the control group (d=0.12).

#### Physical Activity

Both groups decreased their physical activity during the intervention period. Intervention group participants had a mean decline of 3.36 total weekly METS from the baseline; the control group had a smaller mean decline of 1.03 (d=−0.11).

**Table 2:** Means and SDs for dietary and physical activity outcomes.

Variables	Time point	Intervention (n=32), mean (SD)	Control (n=36), mean (SD)	Effect size d (95% CI)
Fruit servings	Baseline	0.75 (0.76)	0.96 (0.70)	
	Follow up	0.84 (0.79)	0.88 (0.68)	
	Change	0.09 (0.46)	−0.08 (0.57)	0.33 (0.21 to 0.45)
Vegetable servings	Baseline	1.30 (0.99)	1.18 (1.06)	
	Follow up	1.38 (1.08)	1.16 (0.95)	
	Change	0.08 (0.91)	−0.02 (0.79)	0.12 (−0.08 to 0.32)
Combined fruits and vegetable servings	Baseline	2.04 (1.50)	2.14 (1.57)	
	Follow up	2.22 (1.70)	2.04 (1.43)	
	Change	0.18 (1.11)	−0.10 (1.14)	0.25 (−0.01 to 0.52)
Weekly total METs^a^	Baseline	24.55 (21.01)	29.81 (25.14)	
	Follow up	21.19 (21.64)	28.78 (21.04)	
	Change	−3.36 (21.70)	−1.03 (21.01)	−0.11 (−5.12 to 4.89)

^a^METs: Metabolic Equivalent of Task units.

### Patient Evaluation Feedback

At the 3-month assessment, all intervention group participants (n=32) completed survey items to provide feedback on their experiences with the intervention. Results are summarized in Table 3. Among the intervention group, 72% (23/32) viewed the DVD, with 50% (16/32) completing the entire DVD. Of those who used it, 60% did so more than once. More than half (14/23) rated it as easy to use, whereas a third (35%, 8/23) found it neither easy nor difficult. Most reported that it kept their attention at least somewhat (87%, 20/23), and looked professional (96%, 22/23). All the participants (100%, 23/23) stated it did a good job of presenting health information, found nothing offensive in the material, and was culturally appropriate, with only 1 person stating it made her feel uncomfortable. Users found it relevant for them as cancer survivors (91%, 21/23). Overall, they felt it was the right length (83%, 19/23), were satisfied (30%, 7/23), or extremely satisfied with it (61%, 14/23), and would recommend the intervention to others (91%, 21/23).

**Table 3:** Patient evaluation feedback for the intervention group.

Evaluation item		n (%)
**How much time did you spend using the program? (n=32)**		
	None	9 (28)
	5-10 mins	2 (6)
	10-20	5 (16)
	All of it	16 (50)
**What part did you watch? (n=23)**		
	Intro	22 (96)
	Physical activity	19 (83)
	Healthy eating	22 (96)
**How many unique times did you use the program? (n=20)**		
	Unsure	1 (5)
	1	7 (35)
	2-3	7 (35)
	4-5	3 (15)
	More than 5	2 (10)
	missing	3
**How easy or difficult was it to use? (n=23)**		
	Very easy or easy	14 (61)
	In between	8 (35)
	Very difficult or difficult	1 (4)
**How easy was it to see the on-screen text? (n=23)**		
	Very easy	14 (61)
	Easy	8 (35)
	Neither easy or difficult	1 (4)
**To what extent did the program keep your attention? (n=23)**		
	Very much	15 (65)
	Somewhat	5 (22)
	In between, so-so	3 (13)
**How would you rate the professionalism or production value of the program? (n=23)**		
	Very good (like something I’d see on TV)	14 (61)
	Somewhat good	8 (35)
	Very poor (looks unprofessional)	1 (4)
	Did a good job at presenting health information	23 (100)
	Speaks to you as a cancer survivor	21 (91)
	Was nothing offensive or problematic	23 (100)
	The program made me feel uncomfortable	1 (4)
	The suggestions and content were appropriate for someone from your culture and background	23 (100)
**Overall experience with program (n=23)**		
	Extremely satisfied	14 (61)
	Satisfied	7 (30)
	In between	2 (9)
	Dissatisfied	0 (0)
**Would you recommend the program to others? (n=23)**		
	Definitely	14 (61)
	Probably	7 (30)
	Maybe	2 (9)
**How would you rate the length of the program? (n=23)**		
	Too short	1 (4)
	About right	19 (83)
	Too long	3 (13)

Qualitative interviews were also conducted with 12 of the intervention group participants who reported using the intervention. Three key themes emerged from the qualitative analysis of transcripts regarding engagement, content, and usability. Key themes and select quotes are presented below:

Theme 1: The program engaged patients’ interest as cancer survivors *.* Patients liked that it came from a reputable information source, found it to be engaging and interesting, liked the positive, encouraging tone, and found the survivor stories to be inspiring. Participants suggested greater racial, ethnic, and socioeconomic diversity of the survivors who were interviewed, and 1 user did not like that a survivor interview mentioned cancer as a “blessing in disguise.”

Because when you start watching what’s on the DVD, you know, you get very interested. It’s informative. You know, so whatever you watch and memorize, it motivates you.

*I think the whole DVD is a very positive, you know, approach, and it had a nice balance of survivors and then professionals.*

Theme 2: Patients made suggestions for adding specific physical activity and healthy eating content. Nutrition and physical activity information was perceived to have been presented with an appropriate level of detail, and participants appreciated the focus on setting small goals and how to incorporate changes into their daily routines. They found it to be motivational and a good reminder of what their goals should be. In terms of preferences not included, patients requested more information on how fruits and vegetables and activity help the body physiologically, specific recipes, more exercises to perform, and how to tailor exercise to various needs such as living in urban areas or for older persons.

It’s very good for survivors, you know? It—it helps us to learn how to control our eating habits, especially, you know, when you’re not used to eating that healthy. I liked how the CD was set up, how—if I remember correctly, showing pictures, and not just somebody lecturing you, but they showed you, it was more interactive, showing you pictures of things and—that was helpful.

*Take it to the next level. That was my biggest complaint about it, that I wanted more.*

Theme 3: Patients liked the interactive structure and suggested usability improvements. Patients reported that the self-assessments were engaging and helped determine their current eating and activity habits. Patients generally found the menu structure easy to use on screen, but wanted it to be easier to go back and review sections. Patients also found it difficult to access the additional resources listed as they had to write them down. Patients noted they would want to have a follow up with greater detail and more specifics, with some noting they would like to have it available in a mobile app version they could access more readily on a mobile phone or tablet.

I was curious to see, you know, with the questions they were asking me, of where that—to go on to find out where I stood.

*They didn’t seem in depth enough for me to—you know, again, I think the disconnect I have is you sit, you watch it, and then you’re left to your own devices. So, if there was something that I could—you know, again, that I could take with me to refer to during the day, I think that would impress me more, you know, impress upon my life more.*

## Discussion

### Principal Findings

This pilot study examined the potential of a theory-based, eHealth intervention designed to assist adult cancer survivors make improvements in healthy diet and physical activity. Results indicate that recruitment and retention was feasible for this older adult survivorship population and that they had high interest in the intervention. Results on behavior change outcomes should be interpreted with caution as the study was not powered to detect reliable differences. Observed power for the effect sizes ranged from .06 to .21. Findings indicate small effects on dietary outcomes, primarily fruit intake, and suggest that additional modifications would be necessary to increase efficacy of the physical activity component.

Results should be interpreted with regard to feasibility of pursing a larger powered trial. The following criteria were assessed as indicators of whether to pursue follow-up work: recruitment of the target sample size in the allotted timeframe, acceptance rate of at least 50% [40], retention of at least 80% [40], at least small effect sizes (d=0.2) on primary outcomes, minimal adverse events, and patient report of interest in and acceptability of the intervention. In light of these criteria, recruitment goals were met within the allotted time frame of 7 months. The study intended to recruit equal numbers of prostate and breast cancer survivors, but a loss of clinic staff resulted in no new survivorship visits for men during the recruitment period. Thus, unfortunately, we do not know how men would respond to the intervention. Consent rates among those eligible were good (53%) and consistent with or higher than diet and activity studies among cancer survivors [41]. Response bias is always a possibility, but given our inclusion criteria, the identified and final sample was likely to differ little by demographics or treatment characteristics. Follow-up rates in the control group met the criteria (86%), but could slightly be improved in the intervention group (73%). No adverse effects were reported by the participants. The majority of the intervention group (72%, 23/32) used the DVD and rated it highly in terms of engagement and usability. Interviews with participants indicated that they found it to be helpful.

For fruit and vegetable intake, a small effect was observed for increased consumption for the intervention as compared with the control group at 3-month follow up, primarily attributable to increased fruit intake (d=.33) versus vegetables (d=0.12). The mean difference between intervention and control in combined fruit and vegetable score would be comparable with about 0.28 standard servings per day. Intensive, multisession telephone-counseling studies conducted with survivors have generally observed increases of 0.5-0.9 servings a day at the 3-month follow up [17,18]. Thus, the results here make sense for a low-contact intervention. Nevertheless, effects on fruit and vegetable intake would meet the criteria for pursuing a larger trial, albeit with greater attention to intervention intensity to further improve results.

The results did not meet the criteria for physical activity. Surprisingly, declines in METs were observed in both intervention and control groups with a slightly greater decline in the intervention group compared with the control. We investigated additional analyses to provide further insight into this finding. As participants only had to have at least one risk factor (meaning some were already active) we analyzed results by baseline activity and found an interesting trend—those who were already physically active and who used the intervention had less of a decline in activity compared with those who did not use the intervention; participants who did not meet the physical activity criteria at baseline showed a trend for greater increase in activity compared with those who did not use the intervention (Multimedia Appendix 1). These follow-up findings were similar to the findings of Pinto et al [42], who found that those who were more active at the baseline regressing to the mean at follow up. It should also be noted that in this study, the control group reported more physical activity at the baseline than the intervention group. Other studies have also observed over-reporting of physical activity at the baseline [43], which increases the difficulty of observing changes at follow up. It is noteworthy that study follow-ups were conducted solely during the winter months in the Northeastern United States, such that seasonality may potentially explain the overall mean decreases in activity for an older survivorship population. The intervention’s focus on low-impact activities such as walking could also have led participants to decrease their activity during the winter months.

In terms of informing a larger trial, the recruitment plan and follow up generally went well, a small effect was observed on fruit and vegetable intake, and patients liked the program. Nevertheless, results for physical activity were disappointing. A number of improvements would be indicated prior to pursuing further work. In terms of recruitment, contacting patients via telephone appeared a difficulty given that few people now answer their phones. Although a more time-intensive method, it may be necessary to meet patients first in person to offer study enrollment versus on telephone or through email. Nowadays, we employ an “on-call” research assistant who can quickly come to the clinic when a provider identifies an interested patient. Follow up should be extended to 6 months or a year as time and funding limited the period of study here. In terms of measurement, while standard measures of diet and activity were used, these rely on self-report and recall. Using multiple 24-hour recalls (ie, ASA24) would also improve assessment of dietary practices, and which are now available on the Internet through the National Cancer Institute (NCI) [44]. Measurement of physical activity, in particular, would benefit from use of accelerometers and mobile heart rate monitors now available that can more accurately capture additional activities such as strength training. In addition, depending on location, physical activity studies should account for seasonality by conducting the intervention period over a longer time frame and by stratifying by season of recruitment.

### Conclusions

In terms of behavioral endpoints, promoting lifestyle changes among cancer survivors remains a challenge. Studies of fruit and vegetable consumption indicate that it has tended to be an easier behavior to improve than physical activity, likely due to a number of factors; it does not require much additional time or scheduling changes, offers immediate reward, has few if any contraindications due to comorbidities, does not result in physical discomfort, and does not require large increases in knowledge or skills [21]. Physical activity, on the other hand, has been a particularly difficult intervention endpoint in cancer survivorship. A comprehensive review of physical activity interventions in cancer populations found that no studies reported 75% or greater adherence to the 150 min per week guideline, even when they used multisession counseling and supervised training sessions [21]. The most common barriers survivors report are being “too busy” and lack of “willpower,” factors which predict level of activity [45]. As survivors are also concerned about safety and comorbid medical conditions, combining introductory in-person demonstrations [46] along with an interactive self-guided program would better address barriers related to self-efficacy, motivation, and time. Achieving and sustaining robust behavior change will likely require further contacts and specific goal setting and monitoring, enhancements that have been linked to increased behavioral adherence [21,47]. Interviews with participants indicated interest in having the resource available via a mobile platform that would enable additional features such as tracking and goal setting. Indeed, a mobile phone app modality to present information, combined with automated tailored text messaging, to provide ongoing intervention components may be one strategy to integrate these features into an updated intervention. Recent reviews have called for interventions that can be more readily disseminated to a more diverse range of survivors beyond those who can attend inperson sessions at major cancer centers [21]. This study provided important insights that can be integrated into a more intensive mobile-based intervention, which is planned in a future trial.

## Appendix

Multimedia Appendix 1 Follow up analysis and example of tailoring algorithm.

## Abbreviations

DVD: digital video disk
HRQoL: health-related quality of life (HRQoL)
METs: Metabolic Equivalent of Task units
