# Supplementary material for: An eHealth Intervention to Increase Physical Activity and Healthy Eating in Older Adult Cancer Survivors: Summative Evaluation Results
Source: JMIR Cancer. 2017 Mar 1;3(1):e4. doi: 10.2196/cancer.6435 (PMC5392211; doi:10.2196/cancer.6435)
Supplement: Multimedia Appendix 1 [file cancer_v3i1e4_app1.pdf]

Figure 2: Mean Changes in METs among DVD Users and Nonusers by Baseline Physical Activity (intervention group only)

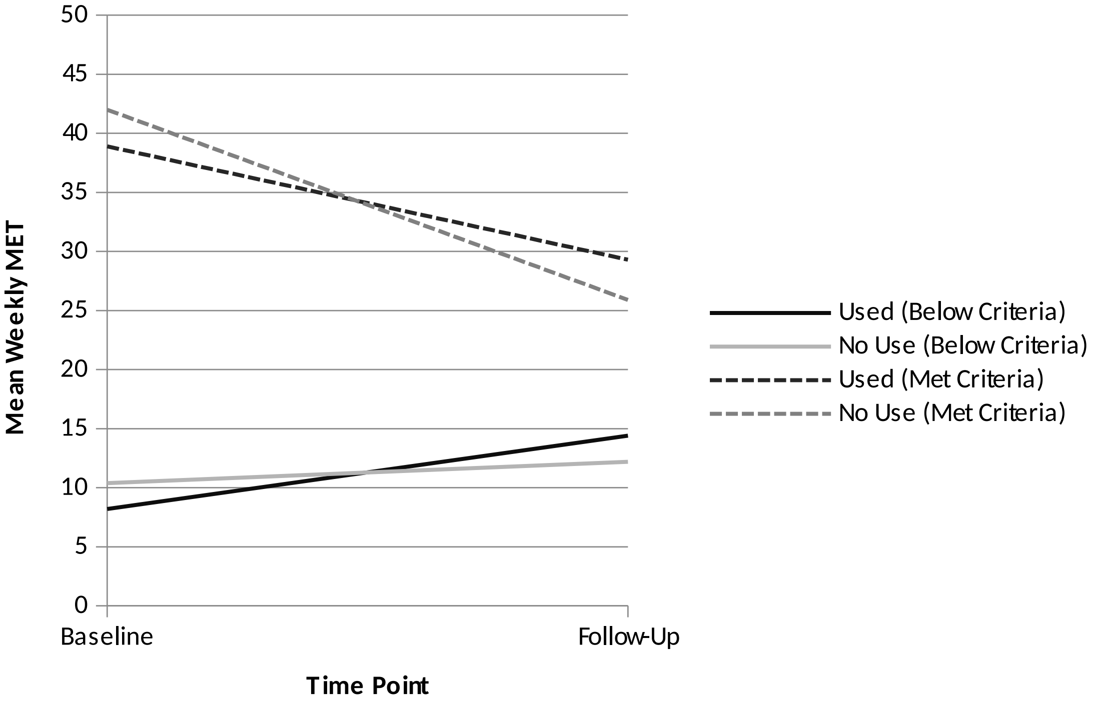

Supplemental Table: Tailoring constructs and feedback.

• Constructs indicated in Blue<br>• **Bold indicates Text on Screen (which is also narrated)**<br>• *Italics indicates voiceover. Voiceovers accompanied by related videos, animations or pictures.*		
Fruit /Vegetable Assessment<br>(note this is only for the tailoring NOT the outcomes)<br><br>**About how many servings of fruit and vegetables are you eating each day?**<br><br>**(Examples of 1 serving are: One medium apple, a banana, an ear of corn, 2 cups of lettuce, or 12 baby carrots.)**	❑ **I am eating *less than* 5 servings each day**	*You said weren't quite having 5 servings every day of fruit and vegetables. That's ok. It's easier than you think to get there.*<br>*There are many reasons why people don't eat fruits and vegetables. For some people aren't used to eating them, or maybe you don't think you like them. Some people say they've looked at prices and found them expensive, or don't have time to get to the store. Thinking about some of your own reasons will help you see if you want to make this a goal for yourself.*<br>*This program will show you easy ways for you to add more fruits and vegetables to what you eat every day.*
	❑ **I am eating *more than* 5 servings each day**	*You said you were eating at least 5 servings every day of fruit and vegetables. That's great! Keep up the good work.*<br>*If 5 is already comfortable for you, you may want to consider increasing your servings to 9 each day, which may be even more helpful to your health.*<br>*This program will show you ways to continue healthy eating and give you some useful tips for adding even more fruit and vegetables to what you eat every day.*
Readiness<br><br>**How *ready* are you to start eating at least 5 or more fruits and vegetables each day?**	❑ **I plan to start in the next 30 days**	*You said you were ready to start in the next 30 days. You're on your way!*<br>*Small steps can lead to lasting changes. During the next month take at least one small step toward having more fruits and vegetables.* ***Check off one thing you think you can do:***<br>❑ **Add a piece of fruit to a morning or afternoon snack**<br>❑ **Try one new fruit or vegetable you haven't had before**<br>❑ **Try one healthier option when you eat out at a restaurant**<br>❑ **Look for new recipes that use vegetables in them**<br>❑ **Make one day each week 'meat free'**
	❑ **I plan to start in the next 6 months**	*You said you were ready to start in the next 6 months. This means you are thinking about making some changes.*<br>*You are aware of benefits of making changes to how you eat, but may have some doubts. Some people aren't quite sure what to do or may be worried they have to give up things they like. In this stage it's easy to keep putting off taking action. This program will help you take the next step by showing you small,*

		easy changes to get started.
	❑ **I have no plans to start right now**	*You said you didn't have any plans to start eating more fruits and vegetables right now. What we eat is very personal so it can seem like quite a change. You may not have thought too much about what you eat. Or, you may be thinking that changing what you eat is too complicated.*<br>*We encourage you just to be open to learning some new things. Some things to think about are:*<br>• *How might eating healthy foods help me feel better?*<br>• *How does it help people who've had cancer?*<br>*Once you know more about what healthy eating means, you can decide for yourself if it's something you may want to try*
Benefits	**Think about these reasons for eating healthy foods and check off the most important one for you:**	❑ **It will give me energy**<br>❑ **It will help me keep a healthy weight**<br>❑ **I will worry less about my health**<br>❑ **I will feel better**<br>❑ **I would like to avoid getting another cancer**<br>❑ **I would be proud of myself**<br>❑ **I would be able to do more**<br>❑ **I would be happier about how I looked**<br>❑ **It would help my blood pressure, diabetes, or heart condition**
Goal Setting	**Which option do you think you could try this week?**	❑ **Try vegetable stir fry, vegetable soup, vegetable fajitas, veggie wraps, veggie burgers, or pasta primavera.**<br>❑ **Order a side of veggies with your meal, or pick three or four veggie sides and create your own vegetable plate.**<br>❑ **If going for pizza, have a salad to start and then add vegetables to the pie.**
Barriers	❑ **I and/or my family doesn't like vegetables. How do I get them to eat more?**	*This can be hard when we are eating with others with different likes and dislikes. Try adapting your usual recipes and adding some vegetables. Mac and cheese with broccoli, or pasta sauce with extra vegetables are easy options to start.*
	❑ **Fresh fruits and vegetables go bad before I can use them. What can I do to minimize waste?**	*It's important to find a way that works for you and within your budget. It doesn't have to be expensive. One tip is to try frozen vegetables. They have just as many nutrients and you can use as much as you need each time. Some fruits last longer than others. Apples or pears are good options.*
	❑ **Fruit and vegetables don't fill me up.**	*Many people think this, but actually foods that have a lot of water or fiber and little fat are usually low in calorie density. They will help you feel full without an unnecessary amount of calories.*

Knowledge<br><br>**Which would you like to hear more about?**	❑ **Recommendations about alcohol**	*Many people ask about alcohol intake. Many studies have found a link between alcohol intake and risk of some primary cancers, including cancers of the mouth, pharynx, larynx, esophagus, liver, breast, and probably colon cancer. In individuals who have already received a diagnosis of cancer, alcohol intake could also affect the risk of new primary cancers of these sites. Given that alcohol has been found to have some protective effects for cardiovascular health, whether to consume it depends upon hereditary disposition and risk of recurrence versus risk of cardiovascular disease. It is generally recommended that women have no more than one alcoholic drink per day and men no more than two. One standard drink means either 6oz of wine, one beer, or one once of liquor.*
	❑ **Red meats**	*Some people ask if they should avoid meats. Large amounts of red meat and processed meats (like bacon, hot dogs, and deli meats) have been linked with increased risk of colorectal, prostate, and stomach cancers. Choose whole cuts of lean meats such as chicken or turkey and limit red meat (such as beef, pork, and ham) to no more than once per week. Research is needed to determine if growth hormone-free and antibiotic-free meats and poultry confer the added benefits that their advocates claim. Regardless, they are safe to add to the diet.*
	❑ **Organic foods and vegetarian diets**	*People also often ask about organic foods and vegetarian diets. There isn't any direct evidence that organic or vegetarian diets help reduce the risk of cancer coming back when compared to diets high in vegetables, fruits, and whole grains and low in red meats. But vegetarian diets can be good for you because they tend to be low in saturated fat and high in fiber, vitamins, and phytochemicals. For these reasons, American Cancer Society Guidelines for cancer prevention recommend eating fruits and vegetables over processed and red meats.*

**Physical activity assessment**<br><br>**For how much time in the past 2 weeks have you done any moderate physical activity?**	❑ **I did at least 2.5 hours each week**	*You said that you were getting at least 2.5 hours every week of moderate activity. That's great! You're getting the amount of activity that is suggested for cancer survivors. This program will give you information about why activity is important for cancer survivors and maybe some tips to help you keep meeting your goal.*
	❑ **I did some, but less than 2.5 hours**	*You said that you were getting some moderate physical activity each week. That's Good! Increasing your weekly physical activity would give you even more benefits. Is there a way you can add a few more minutes of activity each week? This program will give you some tips and suggestions about how to get there.*
	❑ **I didn't do any moderate physical activity**	*You said that you weren't getting any physical activity at a moderate level each week. Many things can get in the way of being active, such as being busy, tired, or having a condition that limits your movement. Even if you have limitations, there are many things you can still do. Even a little bit of activity can be helpful. Increasing the time and level of your activity can improve many areas of your health. It can help your heart, blood pressure, stress, weight, and energy. Talk to you doctor to see what exercises you are able to do. Keep thinking about how you can get started. There are easy ways to start and this program will show you how*
**Readiness**<br><br>**The American Cancer Society suggests that people who've had cancer get at least 2.5 hours of moderate physical activity each week.**<br>**How ready do you feel to start doing this?**	❑ **I'm already getting this much activity**	*You're getting the amount of activity that is suggested for cancer survivors. That's really great. The most important point for someone like you is to make a plan for maintaining this level of activity. We all have times when we get busy or stressed, which makes it harder to be active. It helps to plan for these challenges and have a plan for working through them. What are your challenges to being active and how can you prepare for them?*
	❑ **I'm thinking about meeting this goal in the next 30 days.**	*You said that you were planning on meeting this goal in the next 30 days. That's great!*<br>*You can get started by setting small goals for yourself. This program will give you more information about how to be more active.*
	❑ **I'm thinking about meeting this goal in the next 6 months**	*You said that you were planning on meeting this goal in the next 6 months. It can be a struggle to get started but it is great that you are thinking about it.*<br>*Keeping active, even a little bit, is important for your health and will help you feel better in many*

		ways. This program will give you tips on how to get started

	❑ **I don't plan to start getting this much activity right now**	You said that you were not planning to start getting moderate activity right now. Even if you have a condition that limits your movement, there are many activities you can still do.<br>Talk to your doctor or a physical therapist about suggestions that are OK for you. Keeping active, even a little bit, is important for your health and will help you feel better in many ways.
Benefits	**These are some reasons people want to be active. Check off the one most important for you:**	Whenever we make a choice, there are 'pros' or benefits of the change and 'cons' or drawbacks of making the change.<br>Thinking more about the *benefits* of making a change – the good things we want from it – rather than the drawbacks – how it would be difficult- will help you meet your goals. These are some reasons people want to be active. Check off the one most important for you:<br><br>❑ **Will give me more energy**<br>❑ **Will help me feel I am taking care of myself**<br>❑ **Will help me control my weight**<br>❑ **I would feel proud of myself**<br>❑ **I would feel less stressed if I exercised regularly**<br>❑ **Exercising puts me in a better mood for the rest of the day**<br>❑ **I would feel more comfortable with my body if exercised regularly**
Barriers<br>**Which one of these situations is something you think you'll come across?**	❑ **I'm too tired**	*Many treatments from cancer can leave you with lingering feelings of being tired. If you're too tired, exercise may actually help you! It gives you that boost of energy you may need to get through the day. Try exercising in the morning- you'll notice that you'll feel better the rest of the day.*

	❑ **I'm too busy, I don't have the time**	*Few people really have extra time in our schedules, but if it is a priority most people can find time each week.*<br>• *To make it easier, try being active for shorter amounts of time. For example, instead of exercising for 30 minutes straight, you can take 10 minute walks, 3 times a day. It all counts!*<br>• *Take a walk on your lunch break, around the parking lot or neighborhood. Or stay a few minutes longer after work before you go home and get some walking in.*<br>• *Find a friend or family member to walk with you. It's easier to do it with somebody, plus you can visit and get your activity in at the same time.*
	❑ **I don't enjoy exercising**	• *Don't think of getting activity as "exercise." There are many enjoyable and easy ways of being active that don't involve a gym. It doesn't have to be hard or painful!*<br>• *Try different activities each day. If you walk today, try yoga , a class, or an exercise video at home tomorrow. Tell yourself how good you'll feel when you are done!*
	❑ **I have responsibilities that keep me at home.**	*For many people with families and busy schedules it can be hard to get time away. You don't have to go to a gym or even outside to be active though! For instance, buy an exercise video. There are many fun programs you can do right at home. Even some of the game systems have activity games that help keep you on track. It can also be helpful to get your family involved too. If it's something you do together, you're more likely to keep it going.*
	❑ **I worry about hurting myself.**	• *Always warm up before and cool down after you exercise.*<br>• *Don't overdo it! If you feel dizzy or get tired while you're exercising, your body is trying to tell you to take a break. So, listen!*<br>• *Get approval from your healthcare provider first.*

Goal Setting	**Pick at least one step can you do in the next 2 weeks:**<br>❑ **Read more online about ways to be active**<br>❑ **Buy a pedometer to track my daily steps**<br>❑ **Go for a 10 minute walk at least once**<br>❑ **Get a smart phone app to track my activity**<br>❑ **Talk with a friend about my goal of getting more activity**	*We've mentioned that the key to being more active is starting with small changes. Pick at least one step can you do in the next 2 weeks.*
Knowledge<br><br>Which would you like to learn more about?	❑ **Flexibility and chair exercises**	*Some people who haven't been active may not feel comfortable at first or wonder if they should be feeling how they are. Here's what to expect….* **Video demonstration at PT gym**.
	❑ **Getting started with moderate activities**	*In this section we'll show you some ways to be active, how to do so safely and how to stay on track with your goals.* **Video demonstration at PT gym.**
